# Automated Non-invasive Video-Microscopy of Oyster Spat Heart Rate during Acute Temperature Change: Impact of Acclimation Temperature

**DOI:** 10.3389/fphys.2016.00236

**Published:** 2016-06-22

**Authors:** Nicolle J. Domnik, Elias T. Polymeropoulos, Nicholas G. Elliott, Peter B. Frappell, John T. Fisher

**Affiliations:** ^1^Biomedical and Molecular Sciences, Queen's UniversityKingston, ON, Canada; ^2^Institute for Marine and Antarctic Studies, University of TasmaniaHobart, TAS, Australia; ^3^Commonwealth Science and Industry Research Organization, Agriculture FlagshipHobart, TAS, Australia; ^4^Zoology, University of TasmaniaHobart, TAS, Australia; ^5^Medicine, Division of Respirology, Queen's UniversityKingston, ON, Canada

**Keywords:** oyster, oyster spat, video microscopy, acclimation temperature, heart rate, heart rate variability, aquaculture

## Abstract

We developed an automated, non-invasive method to detect real-time cardiac contraction in post-larval (1.1–1.7 mm length), juvenile oysters (i.e., oyster spat) via a fiber-optic trans-illumination system. The system is housed within a temperature-controlled chamber and video microscopy imaging of the heart was coupled with video edge-detection to measure cardiac contraction, inter-beat interval, and heart rate (HR). We used the method to address the hypothesis that cool acclimation (10°C vs. 22°C—T_a10_ or T_a22_, respectively; each *n* = 8) would preserve cardiac phenotype (assessed via HR variability, HRV analysis and maintained cardiac activity) during acute temperature changes. The temperature ramp (TR) protocol comprised 2°C steps (10 min/experimental temperature, T_exp_) from 22°C to 10°C to 22°C. HR was related to T_exp_ in both acclimation groups. Spat became asystolic at low temperatures, particularly T_a22_ spat (T_a22_: 8/8 vs. T_a10_: 3/8 asystolic at T_exp_ = 10°C). The rate of HR decrease during cooling was less in T_a10_ vs. T_a22_ spat when asystole was included in analysis (*P* = 0.026). Time-domain HRV was inversely related to temperature and elevated in T_a10_ vs. T_a22_ spat (*P* < 0.001), whereas a lack of defined peaks in spectral density precluded frequency-domain analysis. Application of the method during an acute cooling challenge revealed that cool temperature acclimation preserved active cardiac contraction in oyster spat and increased time-domain HRV responses, whereas warm acclimation enhanced asystole. These physiologic changes highlight the need for studies of mechanisms, and have translational potential for oyster aquaculture practices.

## Introduction

Cardiac control is relatively conserved between phyla, despite inter- and intra-species differences; for example, myogenic propagation of cardiac electrical impulses and the excitatory and inhibitory regulation of heart rate (HR) retain common design principles across evolutionarily distant organisms (Koester et al., 1979). Further, species in which cardiac control emerged prior to mammalian evolution, such as oysters, provide a means to examine the origins of physiologic and pharmacologic mechanisms of cardiac control. In addition to these mechanisms, filter-feeding oysters are part of a growing aquaculture or sustainable food futures industry, which is influenced by changing ocean environments due to development, pollution, and climate change (Doney, 2010; The WorldFish Center, 2011; Ruckelshaus et al., 2013)^1^. Thus, an understanding of the physiology and pathology of commercially-farmed species is increasingly important (e.g., aquaculture accounts for 90% of Tasmanian commercial fisheries production Skirtun et al., 2013 and 10,000 tons of oysters are produced annually in Canada^2^) and represents an opportunity for knowledge translation (KT) to industry practices. For example, cardiac control is altered by changes in temperature, which are a routine challenge for the inter-tidal bivalve mollusc, the Pacific oyster (*Crassostrea gigas*; Thunberg, 1793^3^; Quayle, 1969; Newell and Branch, 1980; Bernard, 1983). Factors such as temperature, salinity, oxygenation, and seston/nutrient loads alter also growth rates, influence survival, and affect physiology and health in open aquaculture environments (Newell and Branch, 1980; Bernard, 1983; Helm, 2005).

Oyster spat reflect the early ontogeny of the physiology of cardiac control and represent a unique stage in the oyster life cycle, which is characterized by rapid growth not yet impacted by the energy-demanding process of spawning (Beiras et al., 1995). Commercially-farmed Pacific oysters are routinely housed in hatcheries during their larval and post-larval spat stages, where they are reared in carefully regulated conditions (e.g., consistent temperature, food availability) targeted for optimal growth. This differs from the natural environment of the Pacific oyster, or that of mature, farmed oysters. The transition from hatchery to inter-tidal farm challenges juvenile oysters and may alter growth; however, little is known about the impact of acclimation temperature (T_a_) on cardiac physiology or the adaptive mechanisms/responses available to oyster spat during acute environmental temperature changes.

Previous studies of HR in adult oysters and other bivalves or molluscs have relied on invasive imaging techniques that disrupt the integrity of one of the valves, a stressor that negatively impacts on results (Veitch, 1974; Koester et al., 1979). Limited non-invasive methods have been tested in mature invertebrates (Dieringer et al., 1978; Depledge et al., 1996; Curtis et al., 2000). Although innovative methods for zebrafish electrocardiogram (ECG) also rely on invasive approaches to assess cardiac rate (Liu et al., 2016), promising results have been reported for HR in Atlantic salmon alevins using microscopy (Polymeropoulos et al., 2014). We set out to develop a robust, automated method to non-invasively detect cardiac contraction for HR and heart rate variability (HRV) measurements in oyster spat maintained at different environmental temperatures (10°C vs. 22°C). Application of the method provides an assessment of the cardiac physiology of oyster spat during acute cold-temperature challenge, as well as illustrates the opportunity for KT to define the impact of environmental rearing practices on animal pathology. The latter could provide an early health assessment system, which precedes the use of “sentinel animals” coupled with biosensors (e.g., adult oysters and mussels) (Kramer and Foekema, 2001; Andrewartha et al., 2016) to monitor health in aquaculture environments.

## Materials and methods

### Overview

**Figure 1:**
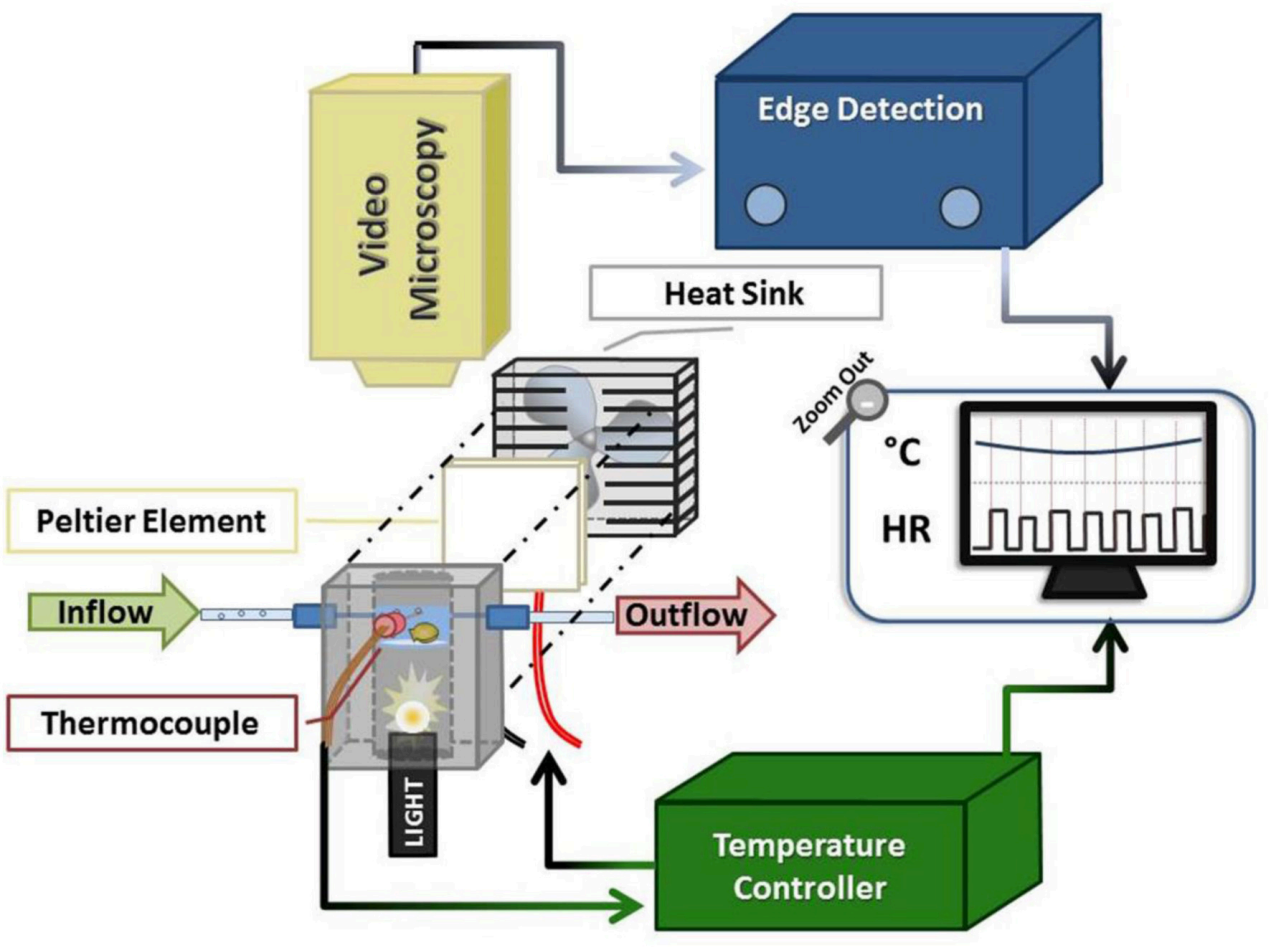
**Schematic of oyster HR detection apparatus**. The system comprised an experimental chamber (gray), a Peltier element (white square + fan), a thermocouple linked to a servo temperature controller, and video microscopy components. The oyster chamber is indicated in light blue (representing the ~1 cm of water in the chamber) within the anodized aluminum block and is positioned above the fiber optic light source housed within the block. See text for additional description.

We designed a customized video-microscopy imaging system to non-invasively visualize Pacific oyster spat heart contractions, while controlling oyster chamber seawater flow and temperature (Figure 1). The system combined off-the-shelf components coupled with customized components employing the principles of Peltier elements (Peltier, 1834; Kubes et al., 1989; Correges et al., 1998). The latter were designed to provide a feedback control system between the chamber temperature and the Peltier element. In our protocols the system provided 2°C step changes in temperature in less than 80 s, although slower and more rapid changes are possible.

**Figure 2:**
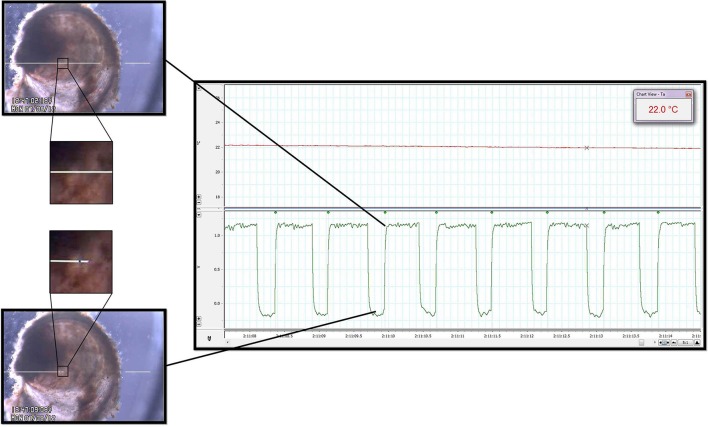
**Heart rate quantification**. Cardiac contraction detection was performed by a commercially available Video Dimension Analyzer (see text) that compared pixel color at an active point (tip of left white line, positioned above oyster; left panels) with pixel color at a reference point (tip of right white line, positioned external to oyster; left panels). The image directly surrounding the spat heart has been magnified to illustrate the color change of the heart at different points during the contractile cycle. The bottom left panel shows the location of the active point at its baseline value during relaxation of the heart. The top right panel shows how, based on gain, cardiac contraction and its resultant pixel color change caused deflection of the active point toward the reference point. The waveforms generated by the edge detector were acquired by the data acquisition system for subsequent analysis (right panel, bottom channel). The time between the leading edges of the onset of each contraction (green waveform detection dots; right panel) provided IBI and HR. Upper channel of the right panel is a record of water temperature as measured by the thermocouple during a period of temperature change between T_exp_. See Video 1 for real-time edge detection imaging.

The system provided clear images of the contracting oyster heart (Figure 2 and Video 1). Contraction was routinely identified in real-time by the automated, commercial edge detection system, while non-detection (due to valve motion, feeding, waste clearance etc.) was easily identified by the experimenter for exclusion. Coupled with the data acquisition software, the edge detection system provided inter-beat interval (IBI; time between consecutive cardiac contractions) and HR for subsequent calculation of HRV responses of spat during acute temperature change. The system also provided visual (video) and quantitative means to assess the incidence of asystole (cessation of cardiac contraction). The oyster spat cardiac contraction detection system and its components (Figure 1) can be obtained and assembled by investigators with moderate effort to provide robust detection for the calculation of IBI and HR.

### Apparatus and software

The experimental set-up consisted of three components: an experimental chamber, a customized electronic temperature-controller, and video microscopy coupled with commercial edge-detection technology (Figure 1).

The experimental chamber was contained within a custom-fabricated 8 × 4.5 × 5 cm anodized aluminum block with a 2.5 cm-diameter bore drilled lengthwise through its center. At a depth of 2.5 cm into the bore (measured from upper block surface) a clear watch-glass was secured and sealed in place. Three small (~1–1.5 mm diameter) ports were drilled into three of the block sides (depth of ca. 2 cm from upper block surface) to allow for the insertion of a thermocouple and seawater inflow/outflow tubes (catheter tubing inserted via permanently-affixed syringe tips).

Seawater was maintained at a depth of ca. 1 cm within the experimental chamber (bounded by the watch-glass and walls of the aluminum bore), with inflow and outflow rates of ~2.5 mL/min (inflow: gravity drip; outflow: peristaltic pump and Pressure Servo Control; Living Systems Instrumentation, Burlington). The oyster spat, which were individually placed atop the watch-glass within the water, were trans-illuminated via a Microlight 150 fiber optic light source (Fibreoptic Lightguides Australia; AIS Optical) contained within the aluminum bore below the watch-glass chamber. A custom servo temperature controller maintained chamber water temperature within ±1°C of each desired experimental temperature (T_exp_) set-point by means of a heat sink (fan)-coupled Peltier element; feedback was obtained via the thermocouple inserted into the center port of experimental chamber.

The trans-illuminated oyster was visualized from above using a microscope (Zeiss Stemi SV6, Zeiss, Germany) coupled with a digital video camera (SONY Hyper HAD Color Video Camera, Japan). The camera video signal was transmitted in real-time to the computer via a video capture device (Roxio Video Capture – Roxio Central Fx/Roxio Central4; 60 fps) connected to edge detection hardware/software (V94 Video Dimension Analyzer, Living Systems Instrumentation, Burlington, Vermont). Edge detection was used to track cardiac motion in real-time by comparison of pixel color at a reference point in the video image (stationary area with no color change over time; see white line on right of Figure 2 and Video 1) with pixel color at an active area of interest (area of cardiac motion as indicated by significant color change over time; see left white line in Figure 2 and Video 1).

Analog edge-detection and thermocouple data were acquired for analysis via a data acquisition system (PowerLab 4/20, LabChart®7 Pro; ADInstruments, Bella Vista, NSW, Australia). High-fidelity periods of detection were routinely obtained and used for analysis, while occasional segments in which edge detection was compromised were excluded from analysis (for example: short periods of active valve movement, excretion of waste). Detection of IBI (see Figure 2, square waveforms) was standardized to the leading edge of the raw edge detection waveforms, and analysis was performed using LabChart's HRV software module with manual inspection of all analyzed data to confirm accurate detection. We adopted a working definition for cardiac asystole as fewer than 45 cardiac contractions within a particular 10 min protocol segment. This definition reflected the observation that periods of asystole presented as a series of increasingly slow, sporadic contractions at a cooler T_exp_, after which complete asystole occurred and was sustained until a certain T_exp_ was reached during the warming arm of the protocol.

### Model: Pacific oyster spat

The juvenile spat stage follows the 15–30 day larval stage in the oyster life cycle, with few studies available compared with mature oysters (Albentosa et al., 1994; Beiras et al., 1995). During the spat stage oysters develop the morphology typical of bivalves, comprising a flat upper (right) valve and domed lower (left) valve joined at an anterior hinge by an adductor muscle. The spat is covered by two mantles, which participate in shell formation and sensation, and possesses two sets of gills, the vascular sites of gas exchange, in the mantle cavity (Quayle, 1969). As closure of the valves requires activation of the adductor muscle, bivalves preferentially keep their valves open unless faced with marked environmental changes, including acutely lowered temperature (Bernard, 1983). The myogenic oyster heart consists of two auricles and a ventricle, which supply a poorly-defined circulatory network that bathes tissues in blood that is returned to the heart via numerous veins; pulsating accessory hearts are also present, supplying circulation to the kidneys (Quayle, 1969). Oyster hearts are regulated extrinsically and intrinsically by neural, biochemical, and environmental/physical (e.g., temperature, pressure, salinity) mediators (Koester et al., 1979).

### Protocol

Triploid *Crassostrea gigas* spat (age: 3.5–4 months, length: 1.1–1.7 mm) were obtained from a commercial hatchery (Shellfish Culture Ltd., TAS; hatchery temperature ~22°C) and acclimated for a minimum of 2 weeks to either 10°C (T_a10_, *n* = 8) or 22°C (T_a22_, *n* = 8). Spat were maintained in aerated housing containers and supplied daily with hatchery-sourced, filtered seawater containing 2–3 million cells/mL of *Chaetoceros I* (TAS) and *Skeletonema pseudocostatum* (SA), then individually transferred into the experimental chamber (placement of right valve surface toward the video-camera unit).

**Figure 3:**
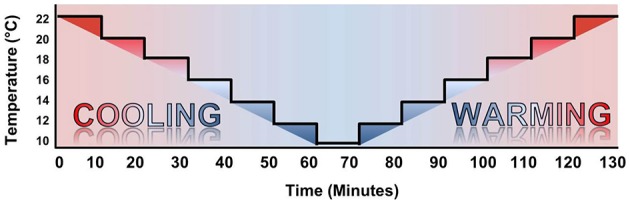
**Temperature ramp protocol**. The temperature ramp was initiated at T_exp_ = 22°C and cooled to T_exp_ = 10°C in 2°C steps with 10 min spent at each T_exp_. Warming steps were identical in timing to the cooling protocol, immediately following T_exp_ = 10°C.

The experiment consisted of a temperature ramp (TR) protocol, where the chamber was set to T_exp_ = 22°C at the onset of the protocol, cooled to T_exp_ = 10°C, and re-warmed to T_exp_ = 22°C. This was done in 2°C intervals, with 10 min at each T_exp_ (Figure 3). The design of the chamber ensured oxygenation and food supply throughout the protocol (as above).

### Analysis

Heart rate, Q10, and time- and frequency-domain HRV analyses were calculated for the data using Excel. During the TR protocol we noted that the spat routinely displayed asystole as a component of the physiologic response to cool T_exp_ and, therefore, quantified the occurrence of asystole for each acclimation temperature (T_a_) group.

Preliminary HRV analysis was applied in the time and frequency domains using scaling factors similar to human HRV, since spat HR was similar to human values. As a result, the scaled time-domain analysis parameter, pNN50, was used across the tested T_exp_. The four time-domain parameters studied were: mean IBI, SDNN (standard deviation of normal IBIs), RMSSD (the root mean square of successive differences of successive IBIs), and pNN50 (the percentage of normal IBIs differing from their preceding IBI by more than 50 ms). Only oysters with active cardiac contraction (i.e., not in asystole) were considered in HRV analyses.

Frequency-domain HRV analysis of IBIs was performed using Kubios software (Kubios HRV version 2.1, University of Eastern Finland, Kuopio, Finland; Tarvainen et al., 2014). Our analysis revealed that oyster spat power spectra failed to display clear peaks aligned with the low- and high-frequency domain peaks used to justify frequency domain analysis in mammalian species (Thireau et al., 2008). As a result, we restricted our HRV analyses to the time domain.

Statistical analyses were performed using SAS V9.4 (SAS Institute Inc., Cary, NC, USA). In order to assess the acute cardiac response to temperature change and the impact of T_a_, which reflects the transition from hatchery to open aquaculture environment, we compared rates of asystole within acclimation groups. Rates of asystole were compared between T_exp_ = 10°C and T_exp_ = 22°C by McNemar's exact test. The difference in asystole rates at 10°C between acclimation groups was tested by Fisher's exact test. Continuous outcomes (HR and HRV parameters) were modeled by the linear mixed effect model estimated by restricted maximum likelihood as implemented by the MIXED procedure of SAS V9.4. The model included fixed effects for acclimation group, temperature, phase (warming vs. cooling), and an acclimation group by temperature interaction to allow comparison of temperature slopes between acclimation groups. The model accounted for the repeated serial measures on spat by including a random effect for spat and allowing for within-spat first order auto-correlated residuals. Q10 values between and within groups were assessed via 2-way ANOVA on ranked data with Shapiro-Wilk and Brown-Forsythe tests. Mean values were reported as mean ± SEM. SEM was not presented in cases where, due to asystole, *n* < 4 for a given group. All tests were two-sided without correction for multiplicity.

## Results

### Cooling-induced cardiac asystole

**Figure 4:**
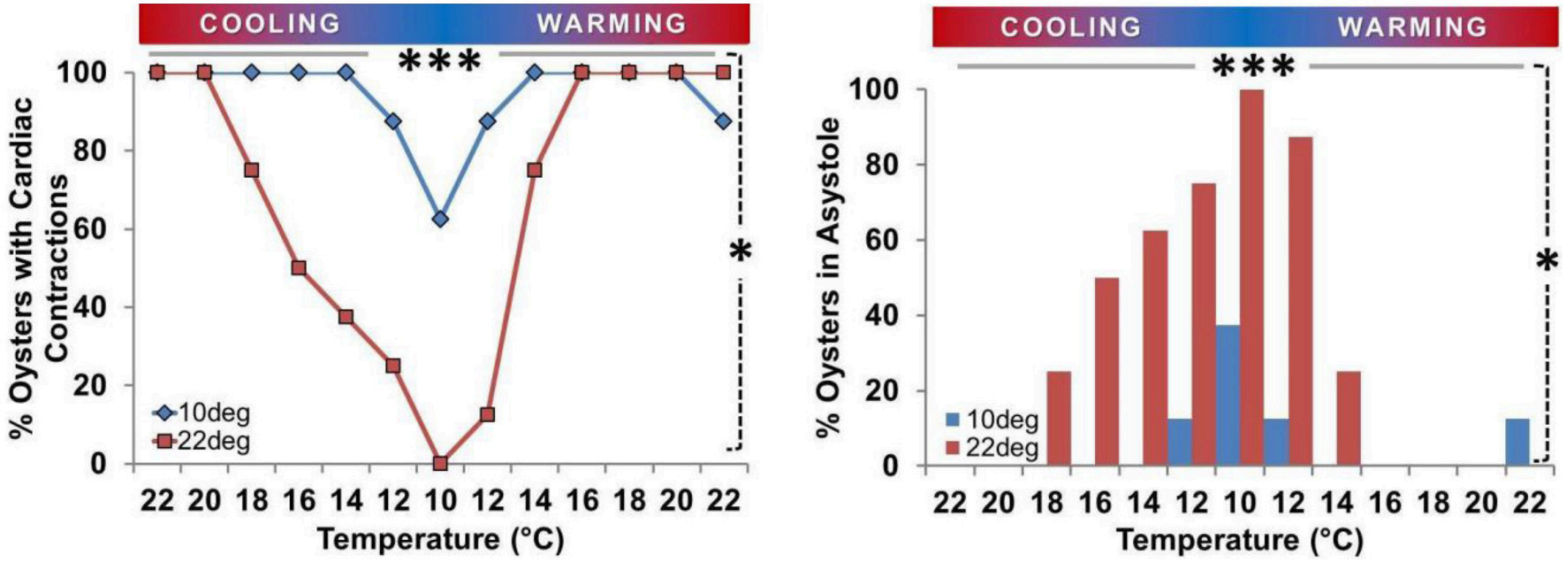
**Prevalence of asystole during temperature ramp protocol**. Proportion of oysters in asystole or maintaining active cardiac contraction (*n*/8) expressed as a percentage. **Left Panel:** Percentage of spat maintaining active cardiac contraction at each T_exp_. Circles/gray bars represent T_a10_ spat; squares/black bars represent T_a22_ spat. **Right Panel:** Percentage of spat displaying asystole at each T_exp_ (< 45 beats/10 min). **Both Panels:** Asystole occurred at low T_exp_. The percentage of spat in asystole was greater at 10° than 22° in T_a22_ spat (*P* = 0.008; horizontal gray bar ^***^), and there was a 62% difference in the prevalence of asystole between T_a22_ and T_a10_ spat at 10° (*P* = 0.026; vertical dashed line ^*^).

Oyster spat routinely adopted cardiac asystole during cooling, and the occurrence of asystole was inversely related to T_exp_ (Figure 4). During cooling from 22°C to 10° C the percentage of spat in asystole increased from 0 to 100% in the T_a22_ group (*P* = 0.008) and from 0 to 38% (*P* = 0.25) in the T_a10_ group. The 62% difference in asystole at 10° C was statistically significant (*P* = 0.026). Figure 4 shows that cardiac activity ceased at higher T_exp_ during the cooling phase than the T_exp_ at which it resumed during warming in T_a22_ spat.

### Spat heart rate responses

**Figure 5:**
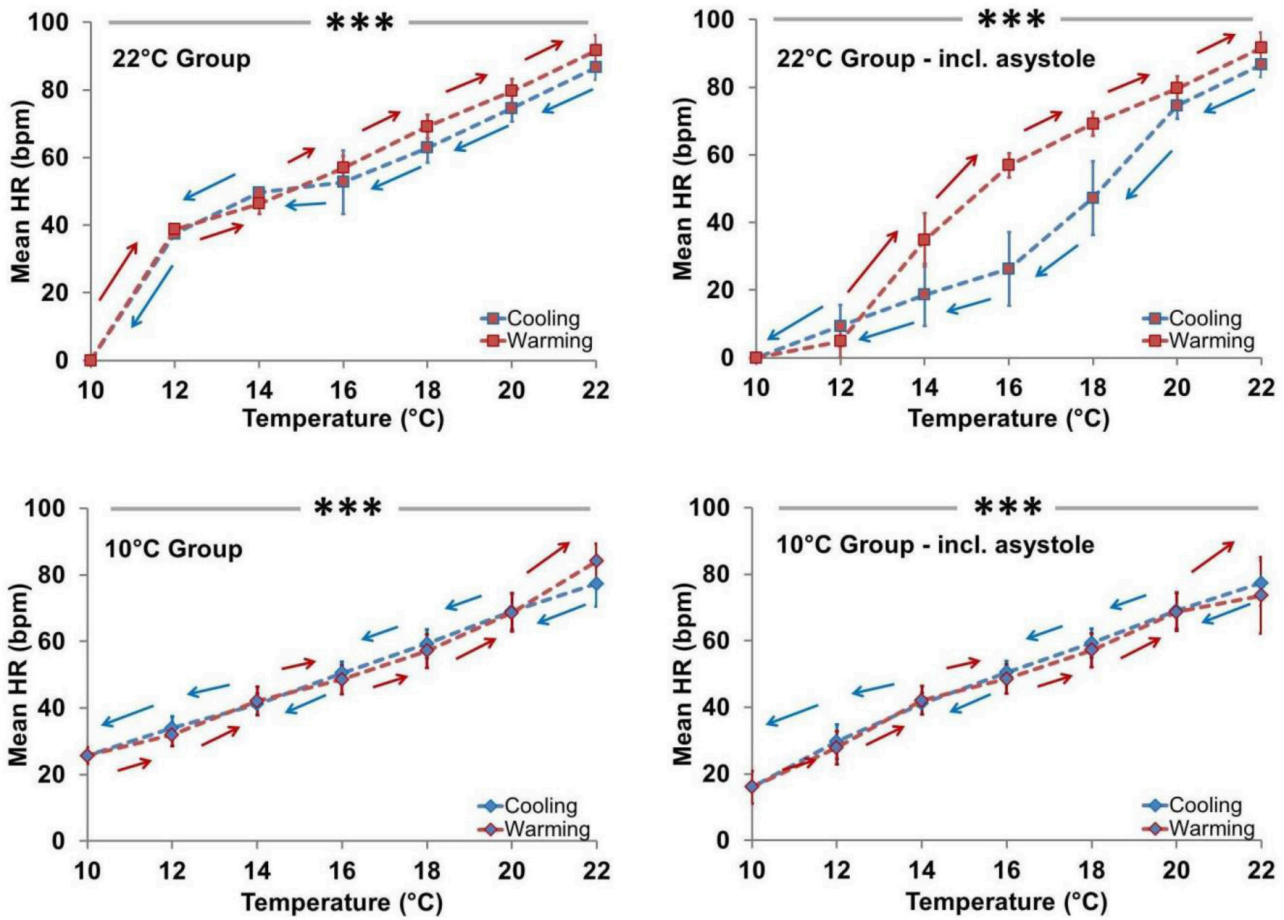
**Effect of temperature (Cooling vs. Warming) on heart rate profile**. HR was directly related to T_exp_ in both acclimation groups independent of asystole. **Left panels:** Mean HR ± SEM of spat showing active cardiac contraction (asystolic spat excluded). In T_a22_ spat, HR decreased at an average rate of 5.5 beats/min per degree Celsius during cooling when asystole was excluded, whereas in T_a10_ spat it decreased by an average of 4.3 beats/min per degree Celsius during cooling when asystole was excluded (both *P* < 0.001; horizontal line ^***^). T_a10_ spat displayed a lower rate of HR decrease during cooling than T_a22_ spat (*P* = 0.032). **Right panels**: Mean HR ± SEM of all spat (asystolic spat included). In T_a22_ spat, HR decreased at an average rate of 7.3 beats/min per degree Celsius during cooling when asystole was included, whereas in T_a10_ spat it decreased by an average of 5.1 beats/min per degree Celsius during cooling when asystole was included (both *P* < 0.001; horizontal line ^***^). T_a10_ spat displayed a lower rate of HR decrease during cooling than T_a22_ spat (*P* = 0.026).

**Figure 6:**
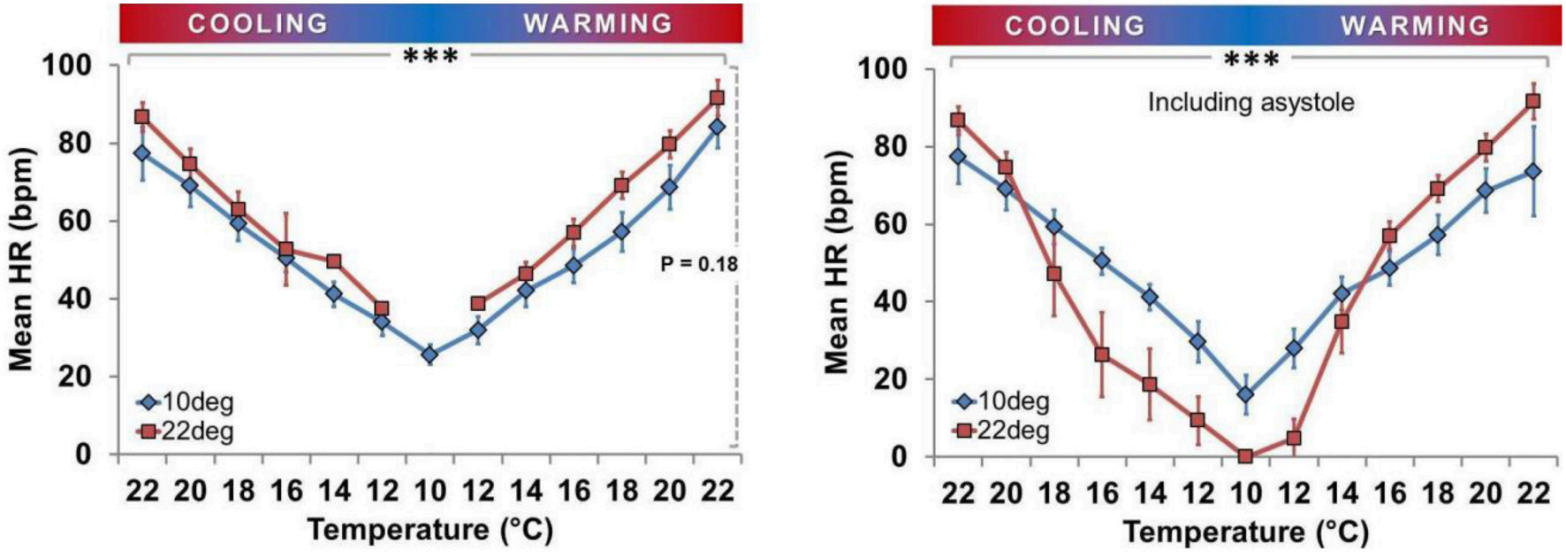
**Effect of acclimation temperature on heart rate during temperature ramp protocol**. HR was directly related to T_exp_ in both groups (^***^horizontal solid bar, *P* < 0.001; squares with dark gray line = T_a22_ and diamonds with light gray line = T_a10_). **Left Panel:** Mean HR ± SEM of spat showing active cardiac contraction only. **Right Panel:** Mean HR ± SEM of all spat, including those exhibiting asystole (HR = 0).

Mean spat HR was positively associated with T_exp_ in both acclimation groups (Figures 5, 6). In T_a22_ spat the average initial HR at 22° was 83.2 beats/min (95% CI, 70.8–95.7) and this decreased by an average of 7.3 beats/min (5.9–8.7, *P* < 0.001) per degree Celsius during cooling (when asystolic spat, as defined in Methods and Materials, were included in calculations as HR = 0). In T_a10_ spat the mean initial HR at 22° was 78.6 beats/min (66.2–91.1) and this decreased by an average of 5.1 beats/min (3.7–6.5, *P* < 0.001) per degree Celsius during cooling (including asystolic spat as HR = 0). The HR of T_a10_ spat at the initial 22° acute temperature was not different from the T_a22_ spat (difference in HR between T_a10_ and T_a22_ = 4.6; −11.7 to 20.9; *P* = 0.57), however, they did exhibit a lower rate of decrease during cooling (difference = 2.2; 0.3–4.2; *P* = 0.026; this included asystolic spat as HR = 0). Excluding spat in asystole, the average decrease in heart rate per degree Celsius while cooling was 5.5 beats/min (4.6–6.3, *P* < 0.001) within T_a22_ compared to 4.3 beats/min (3.7–4.9, *P* < 0.001) within T_a10_ with a significant difference in slopes between acclimation groups (being 1.1; 0.1–2.2, *P* = 0.032).

Figure 5 shows that in the T_a10_ group the T_exp_ specific HR was similar during cooling and warming regardless of inclusion of asystolic spat. However, in the Ta_22_ group, the HR appears to have dropped more quickly during cooling due to the increased proportion of spat in asystole. The cooling and warming patterns in T_a22_ spat did not vary substantially when restricting analysis to spat that maintained active cardiac contraction (i.e., excluding asystolic spat from analysis). Figure 6 (left panel) shows that among spat with active cardiac contraction, T_a22_ and T_a10_ had a similar HR response to T_exp_.

### Impact of acclimation on Q10

Q10 values for mean HR were calculated in the cooling and warming phases for spat in each acclimation group (mean ± SEM). There were no significant differences in Q10 between T_a_ groups, between the warming vs. cooling phases within T_a_ groups, or interactions between T_a_ and phase (Q10 values: T_a22_ – cooling phase: 2.6 ± 0.3, warming phase: 2.4 ± 0.2; T_a10_ – cooling phase: 2.4 ± 0.2, warming phase: 2.7 ± 0.3; all *P* > 0.05, 2-way ANOVA).

### Impact of acclimation on HRV

**Figure 7:**
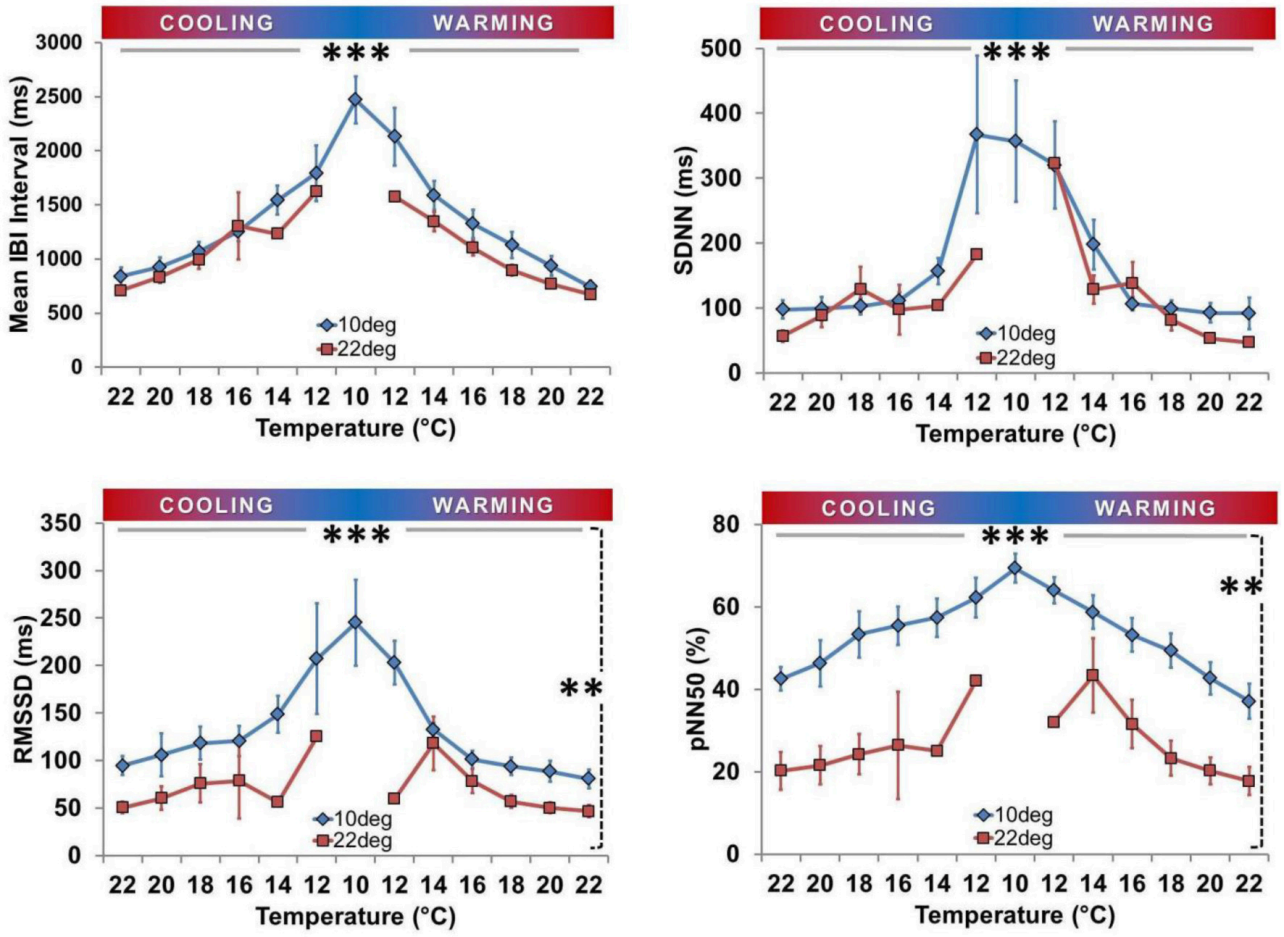
**Time domain heart rate variability analysis**. Means (± SEM) are shown for Inter-Beat Interval (IBI; top left), Standard Deviation of IBI (SDNN; top right), Root Mean Square of Successive Differences of Successive Intervals (RMSSD; bottom left), and Percent of consecutive IBI differing by more than 50 ms (pNN50; bottom right). All four parameters were inversely related to T_exp_ in both groups (^***^horizontal solid line, *P* < 0.01). RMSSD and pNN50 were consistently lower at each T_exp_ for T_a1_0 spat (^**^vertical dashed line, both *P* < 0.001), whereas IBI and SDNN did not vary significantly between groups with respect to acclimation.

There was an inverse relationship between each of IBI, RMSSD, SDNN, and pNN50 with T_exp_ in each acclimation group (after excluding spat in asystole, all *P* < 0.01 for association with T_exp_; Figure 7). At any given T_exp_ RMSSD and pNN50 were significantly higher in the T_a10_ group than the T_a22_ group (both *P* < 0.001), but there were no differences between T_a10_ and T_a22_ groups for IBI (*P* = 0.11) or SDNN (*P* = 0.13; both Figure 7).

## Discussion

We developed an automated, non-invasive, real-time method to detect cardiac contraction and measure HR and HRV in trans-illuminated triploid Pacific oyster spat (*Crassostrea gigas*). The system used video microscopy, edge-detection technology, and a custom-built experimental chamber. We applied the method to assess cardiac responses to acute temperature changes on a background of cool or warm environmental acclimation temperatures. The results suggest that spat may be a promising novel model species to explore conserved mechanisms of HR control. Our method and the results of its first application also have KT implications, since they can be used to assess hatchery practices that influence growth rates, survival, or the physiology of oyster spat prior to placement in the open aquaculture environment (Newell and Branch, 1980; Bernard, 1983; Helm, 2005). The acclimation temperatures of 10°C (T_a10_) and 22°C (T_a22_) reflect aquaculture industry practices, i.e., hatchery temperature (22°C) and a cool temperature that may be encountered upon introduction to an open, temperate aquaculture environment (10°C). We provide the first measurements of the prevalence of asystole, HR response, and time-domain HRV profile of spat exposed to an acute temperature ramp (TR) protocol from 22°C to 10°C to 22°C. Cool temperature acclimation lowered the incidence of asystole in T_a10_ acclimated spat vs. T_a22_ spat and was accompanied by greater HRV. The presence of asystole suggests that oysters reared in the normal hatchery environment (22°C) may be negatively impacted as they are placed into open ocean environments.

### An automated, non-invasive solution to measure juvenile bivalve/small invertebrate heart rate

Our system takes advantage of the relatively stationary nature of bivalves and the translucence of spat shells. The experimental chamber enabled rapid changes in T_exp_ during which cardiac contraction and HR could be assessed relative to T_a_. The system can provide rapid as well as slow temperature changes, which contrasts with existing reports, where experimental chambers are placed or immersed within large, temperature-controlled water baths to make cardiorespiratory or metabolic measurements (Frappell and Mortola, 2000; Polymeropoulos et al., 2014).

Adult oyster hearts have previously been studied *in vitro* and *in vivo* (Greenberg, 1965; Greenberg et al., 1980; Andrewartha et al., 2016), largely through invasive means that disrupt valve integrity to view/record from the exposed heart and negatively influence outcomes (Veitch, 1974; Koester et al., 1979). For example, HR has been measured by incident laser beam reflection from the heart through a several mm diameter hole (Ritto et al., 2001, 2003; Hellicar et al., 2014). Ultrasound stethoscopes (Dieringer et al., 1978) or infrared diodes/phototransistor detectors (Depledge and Andersen, 1990; Depledge et al., 1996; Curtis et al., 2000) have been reported to measure bivalve HR non-invasively. However, the large hardware components make this incompatible with spat (< 2 mm) and these approaches have limited means for verifying that only HR is being detected. By contrast, the thin valves of young spat are amenable to trans-illumination, eliminating physical stressors and attachments, while video microscopy provides an easily verifiable HR signal for the experimenter. Our automated, non-invasive method could be applied to younger or older spat once the limits of microscopic magnification and shell calcification (i.e., animal maturity and size) are defined, or in other species amenable to trans-illumination (e.g., other invertebrates, alevin, Polymeropoulos et al., 2014; eggs, Mortola et al., 2010, 2012). We used the minimum light intensity necessary for cardiac visualization; however, near red light sources could be used to determine/minimize the potential impact of light exposure on spat physiology. The system could also be adapted for use “in the field” in open, aquaculture environments.

### Methodology application: Pacific oyster spat cardiac responses to environmental temperature change

Oyster growth is most rapid at warm temperatures with high food and oxygen availability (Walne and Spencer, 1974); however, the cardiac control phenotype during temperature challenge was not known. As cultured oysters are initially reared in hatcheries providing constant, ideal conditions, our data on the impact of acute changes in variables such as T_exp_, such as those encountered upon initial ocean exposure, suggest ways to measure/improve species health and production.

### Cool temperature acclimation decreases asystole occurrence at low temperatures

The asystole induced during cooling was inversely related to T_exp_, and was more prevalent in T_a22_ spat (8/8 asystolic at T_exp_ = 10°C; Figure 4). Residual beats were generally observed at the onset of a cooler T_exp_, after which complete asystole occurred and was sustained until T_exp_ increased during the warming arm of the TR protocol. This is consistent with a report for the adult blue mussel (*Mytilus edulis*), where asystole occurred at < 8°C if T_a_ = 18°C (Kittner and Riisgard, 2005), although spat achieved asystole at much higher temperatures (all >10°C; Figure 4). Similar to spat, cool-acclimated mussels maintained cardiac activity at lower temperatures than warm-acclimated ones (Pickens, 1965; Braby and Somero, 2006). Thus, lowering the hatchery water temperature in anticipation of placement in ocean oyster beds would reduce periods of asystole and minimize potential reductions in survival or growth. T_a22_ spat became asystolic at warmer T_exp_ during cooling than the T_exp_ at which they regained HR during warming, which may represent a compensatory HR response to clear accumulated metabolites/provide tissues with oxygen and nutrients (Koester et al., 1979; Bernard, 1983). Some conflicting reports exist, with asystole in adult clams (*Noertia ponderosa*) reported to occur at < 5°C but with cardiac activity (HR) only resuming when >15°C (DeFur and Mangum, 1979); this may be attributable to inter-species variation or differences in protocol and acclimation timing. Examining the time-course of acclimation (i.e., resumption of HR in asystolic T_a22_ spat during sustained cool temperature), the ontogeny of the responses in spat of varying ages, and outcomes of growth and time to maturity would be valuable.

Asystole was accompanied by valve closure, which is typically a period of quiescence that may be influenced by food availability, decreased temperature (Koester et al., 1979; Higgins, 1980; Curtis et al., 2000), decreased metabolism with reduced growth (Bernard, 1983), or environmental stressors (Kittner and Riisgard, 2005; Braby and Somero, 2006). Our observations of sporadic residual beats just prior to valve closure due to cooling are similar to reports of the impact of the stressor/pollutant, copper, where increasingly sporadic HR in mussels is present prior to asystole (Curtis et al., 2000). Changes in HR preceding valve closure suggest neural mediation for this phenotype, rather than one in which valve closure, decreased ventilation, and eventual hypoxia trigger decreased HR (Trueman and Lowe, 1971; Koester et al., 1979). Since we maintained equal food availability and water oxygenation, our data for oyster spat likely reflect control mechanisms governed by temperatures lower than the temperature of acclimation.

Consistent with being ectothermic (Widdows, 1973; Braby and Somero, 2006), HR was related to T_exp_ in all spat (Figures 5, 6). The magnitude of the impact of T_a_ on HR depended on whether asystolic spat were included. In contrast to asystole, there was no statistically significant impact on HR between T_a22_ and T_a10_ spat. Interestingly, this was despite what appeared to be a modest tendency toward decreased HR in T_a10_ when asystole was excluded (Figure 6, left panel).

### Cool temperature acclimation increases heart rate variability

Time- and frequency-domain HRV analyses are used to investigate autonomic cardiovascular control (ESC/NASPE Task Force, 1996; Tarkiainen et al., 2005; Thireau et al., 2008). Myogenic molluscan hearts have diffuse pacemaker properties, receive modulatory input from extrinsic cardiac nerves, and are regulated by a combination of neural, biochemical, and environmental/physical (e.g., temperature, pressure, salinity…) mediators (Divaris and Krijgsman, 1953; Trueman and Lowe, 1971; Trueman et al., 1973; Lowe, 1974; Irisawa, 1978; Koester et al., 1979; Ritto et al., 2005). HRV analysis assumes that beat-to-beat variations in HR (i.e., inter-beat intervals, IBIs) are effected by neural modulation (Sandercock and Brodie, 2006; Chattipakorn et al., 2007; Domnik et al., 2012). There is debate on whether to correct for non-cardiac physiologic rhythms (e.g., respiratory sinus arrhythmia) when performing mammalian HRV analyses; however, many studies employ raw HR in the calculation of HRV (Tzeng et al., 2003; Thireau et al., 2008). HRV application to invertebrates is rare, and the relative neural, physical and/or hormonal modulation of HR in oysters has not been fully elucidated (Greenberg, 1965; Quayle, 1969; Mayeri et al., 1974; Liebeswar et al., 1975; Hill and Yantorno, 1979; Koester et al., 1979; Greenberg et al., 1980). Thus, we adopted an approach that performed HRV calculations based on raw HR data, analogous to that employed in mammals.

Few studies have explored HRV in bivalves (Curtis et al., 2000; Ritto et al., 2005). Our analyses revealed none of the defined spikes in post-spectral density (PSD) at specific frequencies as seen in mammals, which is an interesting biologic observation that precluded the application of frequency domain HRV analysis (ESC/NASPE Task Force, 1996; Curtis, 1998; Curtis et al., 2000; Thireau et al., 2008). Mussels have also been reported to have no spikes in PSD (Curtis, 1998; Curtis et al., 2000). The hypothesis of independent control of HR, distinct from ventilation or other physiological functions, is one that deserves further attention. Time-domain HRV analysis (IBI; SDNN; RMSSD; pNN50) applied to oysters not in asystole revealed that all HRV variables were inversely related to T_exp_ (Figure 7). This is similar to findings in mammals, where HRV is inversely related to HR (Billman, 2013; Sacha et al., 2013a,b). It is also reminiscent of the response reported for toxin exposure (e.g., copper), where an inverse relationship between coefficient of variation (variability) and HR was present in mussels (Curtis et al., 2000). Both acclimation groups had low HRV at T_exp_ of 22°C, consistent with low HRV in mussels at control conditions (Depledge et al., 1996; Curtis et al., 2000). This varies from the marked HRV in resting mammals (ESC/NASPE Task Force, 1996; Thireau et al., 2008; Domnik et al., 2012), and may reflect a greater scope for beat-to-beat neural regulation of HR in mammals. T_a10_ spat had increased HRV compared to the T_a22_ group (RMSSD and pN50).

Our findings raise the question of how best to compare and interpret HRV in invertebrates and mammals. Baseline HRV differs between invertebrates and mammals; however, the inverse relationship between HR and HRV remains (Depledge et al., 1996; Kazuma et al., 1997; Curtis et al., 2000; Gong et al., 2004). In mammals, HRV reflects overall autonomic balance and decreased HRV is a negative prognostic indicator (Sandercock and Brodie, 2006; Chattipakorn et al., 2007); one possible interpretation of our findings is that increasing HRV at decreased T_exp_ may suggest the inverse for invertebrate species. We speculate that the adaptive thermal compensatory response to cold T_exp_ and T_a_ may increase HRV in aquatic invertebrates, similar to copper (Curtis et al., 2000) or starvation (Depledge et al., 1996). Further work is required to elucidate the details of this relationship, since T_a10_ spat were less likely to become asystolic at cool temperatures despite exhibiting higher HRV than T_a22_ spat with active HR. The potential predictive power of HRV could be tested using the impact of known stressors (including low food availability and Pacific Oyster Mortality Syndrome (POMS)), which negatively impact on the physiology, growth, and viability of oysters (Widdows, 1973; Duthie, 2014; Andrewartha et al., 2016). The impact of environment on oyster outcomes has been approached through the development and application of habitat suitability indices (HSI); however, outcome measures typically rely on mortality/viability as the primary outcome (Brown and Hartwick, 1988; Theuerkauf and Lipcius, 2016). Evaluation of oyster HR or HRV is the physiologic complement to HSI assessments and has the potential to provide insight into animal health prior to overall mortality measures, as well as to allow the detection of acute environmental changes. Indeed a recent report in the adult oyster suggests that biosensors could monitor HR and health in “sentinel” animals (Andrewartha et al., 2016). This concept is similar to those proposed for other species, such as zebrafish, as biological models or pharmacologic screens (Liu et al., 2016; Rahbar et al., 2016). HRV has been used to explore cardiac regulatory mechanisms, which could be employed to assess cardiac phenotype between triploid and diploid spat or other marine invertebrates. Acclimating oyster spat to lower temperatures prior to release in ocean oyster beds may enhance adaptability to temperature-variable environments, and provide the means to test for early signs of adaptive or compensatory responses to negative environmental changes.

## Conclusions

We developed an automated, non-invasive video-microscopy system to detect cardiac contraction for the real-time measurement of IBI, HR, and HRV in oyster spat that were maintained at different environmental temperatures. The impact of temperature challenge on oyster spat HR and HRV suggests that spat provide a model for conserved mechanisms of cardiac physiology, and also illustrates the potential for knowledge translation to the aquaculture industry. Cooling more readily induced asystole in warm-acclimated spat, although the molecular mechanisms responsible for the ontogeny of the cardiac asystole response remain to be elucidated. Indeed, the energetic cost of such a response may vary at different points in the oyster life cycle, reflecting the strength of the underlying link between cardiac and metabolic control. Our time domain HRV data, the first from oyster spat, require further investigation to determine whether the directionality of HRV changes induced by deteriorating health in mammals is conserved in invertebrate species. Our data suggest that optimization strategies for hatchery temperatures could impact growth/survival in the transition to ocean farms. The method provides a new tool to assess spat resilience to environmental changes or strategies to minimize early spat mortality in open waters (Mann, 1979). Incorporation of measurements of metabolic rate, filtration rate, or growth would provide an integrated analysis of the energetic cost of the observed HR alterations in variably-acclimated spat.

## Author contributions

ND: Design of experiments/hypotheses; methodologic design; performed experiments; analysis/interpretation; wrote and revised manuscript. EP: Assisted with experiments, experimental/methodologic design, interpretation; editing of manuscript. NE: Assisted with experiments, experimental/methodologic design; editing of manuscript. PF: Design of experiments/hypotheses; methodologic design; interpretation; editing of manuscript. JF: Design of experiments/hypotheses; methodologic design; assisted with experiments; interpretation; writing/editing of manuscript.

## Funding

Michael Smith Foreign Study Supplement (National Science and Engineering Research Council, Canada—ND), Canadian Institutes for Health Research (CIHR-MOP 81211—JF), and Commonwealth Science and Industry Research Organization: Agriculture Flagship (Australia—NE).

### Conflict of interest statement

The authors declare that the research was conducted in the absence of any commercial or financial relationships that could be construed as a potential conflict of interest.

## Footnotes

^1^The state of world fisheries and aquaculture. 2012. Rome, Italy, Food and Agriculture Organization of the United Nations.

^2^Farmed Oysters (Sector snapshot). 27-2-2015. Department of Fisheries and Oceans Canada.

^3^http://www.marinespecies.org/aphia.php?p=taxdetailsid=140656

## References

[B1] Albentosa M. Beiras R. Perez Camacho A. (1994). Determination of optimal thermal conditions for growth of clam (*Venerupis pullastra*) seed. Aquaculture 126, 315–328. 10.1016/0044-8486(94)90048-5

[B2] Andrewartha S. J. Elliott N. G. McCulloch J. W. Frappell P. B. (2016). Aquaculture sentinels: smart-farming with biosensor equipped stock. J. Aquac Res. Dev. 7:393 10.4172/2155-9546.1000393

[B3] Beiras R. Perez Camacho A. Albentosa M. (1995). Short-term and long-term alterations in the energy budget of young oyster *Ostrea edulis* L. in response to temperature change. J. Exp. Mar. Biol. Ecol. 186, 221–236. 10.1016/0022-0981(94)00159-B

[B4] Bernard F. R. (1983). Physiology and the Mariculture of Some Northeastern Pacific Bivalve Molluscs. Department of Fisheries and Oceans No. 63, 4–24. Available online at: http://publications.gc.ca/collections/collection_2016/mpo-dfo/Fs41-31-63-eng.pdf

[B5] Billman G. E. (2013). The effect of heart rate on the heart rate variability response to autonomic interventions. Front. Physiol. 4:222. 10.3389/fphys.2013.00222 23986716 PMC3752439

[B6] Braby C. E. Somero G. N. (2006). Following the heart:temperature and salinity effects on heart rate in native and invasive species of blue mussels (genus *Mytilus*). J. Exp. Biol. 209, 2554–2566. 10.1242/jeb.02259 16788038

[B7] Brown J. Hartwick E. (1988). A habitat suitability index model for suspended tray culture of the Pacific oyster, *Crassostrea gigas* Thunberg. Aquac. Res. 19, 109–126. 10.1111/j.1365-2109.1988.tb00414.x

[B8] Chattipakorn N. Incharoen T. Kanlop N. Chattipakorn S. (2007). Heart rate variability in myocardial infarction and heart failure. Int. J. Cardiol. 120, 289–296. 10.1016/j.ijcard.2006.11.221 17349699

[B9] Corròges P. Bugnard E. Millerin C. Masiero A. Andrivet J. P. Bloc A.. (1998). A simple, low-cost and fast Peltier thermoregulation set-up for electrophysiology. J. Neurosci. Methods 83, 177–184. 10.1016/S0165-0270(98)00079-X 9765131

[B10] Curtis T. M. (1998). The Modes of Action of Toxicants on the Cardiac Physiology of the Blue Mussel, Mytilus Edulis, and the Common Shore Crab, Carcinus Maenas. University of Plymouth, Plymouth, England.

[B11] Curtis T. M. Williamson R. Depledge M. H. (2000). Simultaneous, long-term monitoring of valve and cardiac activity in the blue mussel *Mytilus edulis* exposed to copper. Mar. Biol. 136, 837–846. 10.1007/s002270000297

[B12] DeFur P. L. Mangum C. P. (1979). The effects of environmental variables on the heart rates on invertebrates. Comp. Biochem. Physiol. 62A, 283–294. 10.1016/0300-9629(79)90058-6

[B13] Depledge M. H. Lundebye A.-K. Curtis T. Aagaard A. Andersen B. B. (1996). Automated interpulse-duration assessment (AIDA): a new technique for detecting disturbances in cardiac activity in selected macroinvertebrates. Mar. Biol. 126, 313–319. 10.1007/BF00347455

[B14] Depledge M. H. Andersen B. B. (1990). A computer-aided physiological monitoring system for continuous, long-term recordings of cardiac activity in selected invertebrates. Comp. Biochem. Physiol. 96A, 474–477. 10.1016/0300-9629(90)90664-e

[B15] Dieringer N. Koester J. Weiss K. (1978). Adaptive changes in heart rate of *Aplysia californica*. J. Comp. Physiol. 123, 11–21. 10.1007/BF00657339

[B16] Divaris G. A. Krijgsman B. J. (1953). Contractile and pacemaker mechanisms of the heart of molluscs. Biol. Rev. 30, 1–39.

[B17] Domnik N. J. Seaborn G. Vincent S. G. Akl S. G. Redfearn D. P. Fisher J. T. (2012). OVA-induced airway hyperresponsiveness alters murine heart rate variability and body temperature. Front. Physiol. 3:456. 10.3389/fphys.2012.00456 23227012 PMC3514704

[B18] Doney S. C. (2010). The growing human footprint on coastal and open-ocean biogeochemistry. Science 328, 1512–1516. 10.1126/science.1185198 20558706

[B19] Duthie I. (2014). Best Practice Guide for Tasmania Oyster Producers: Biosecurity and Disease Preparedness, with a focus on Pacific Oyster Mortality Syndrome. Tasmania, AUS, Cradle Coast NRM.

[B20] ESC/NASPE Task Force (1996). Heart rate variability: standards of measurement, physiological interpretation and clinical use. Task Force of the European Society of Cardiology and the North American Society of Pacing and Electrophysiology. Circulation 93, 1043–1065. 10.1161/01.CIR.93.5.1043 8598068

[B21] Frappell P. B. Mortola J. P. (2000). Respiratory function in a newborn marsupial with skin gas exchange. Respir. Physiol 120, 35–45. 10.1016/S0034-5687(99)00103-6 10786643

[B22] Gong H. Jr. Linn W. S. Terrell S. L. Clark K. W. Geller M. D. Anderson K. R.. (2004). Altered heart-rate variability in asthmatic and healthy volunteers exposed to concentrated ambient coarse particles. Inhal. Toxicol. 16, 335–343. 10.1080/08958370490439470 15204749

[B23] Greenberg M. J. (1965). A compendium of responses of bivalve hearts to acetylcholine. Comp. Biochem. Physiol. 14, 513–539. 10.1016/0010-406X(65)90224-0 14314989

[B24] Greenberg M. J. Roop T. Painter S. D. (1980). The relative contributions of the receptors and cholinesterases to the effects of acetylcholine on the hearts of bivalve molluscs. Gen. Pharmacol. 11, 65–74. 10.1016/0306-3623(80)90013-0 7364205

[B25] Hellicar A. D. Rahman A. Smith D. Smith G. McCulloch J. (2014). A neural network and som based approach to analyse periodic signals: application to oyster heart-rate data, in IEEE International Joint Conference on Neural Networks (IJCNN) (Beijing), 2211–2217.

[B26] Helm M. M. (2005). Cultured Aquatic Species Information Programme. Crassostrea gigas. Rome: FAO Fisheries and Aquaculture Department [online].

[B27] Higgins P. J. (1980). Effects of food availability on the valve movements and feeding behaviour of juvenile *Crassostrea virginica* (Gmelin). I. Valve movements and periodic activity. J. Exp. Mar. Biol. Ecol. 45, 244.

[B28] Hill R. B. Yantorno R. E. (1979). Inotropism and contracture of the aplysiid ventricles as related to the action of neurohumors on resting and action potentials of molluscan hearts. Am. Zool. 19, 145–162. 10.1093/icb/19.1.145

[B29] Irisawa H. (1978). Comparative physiology of the cardiac pacemaker mechanism. Physiol. Rev. 58, 461–498. 347472 10.1152/physrev.1978.58.2.461

[B30] Kazuma N. Otsuka K. Matsuoka I. Murata M. (1997). Heart rate variability during 24 hours in asthmatic children. Chronobiol. Int. 14, 597–606. 10.3109/07420529709001450 9360026

[B31] Kittner C. Riisgard H. U. (2005). Effect of temperature on filtration rate in the mussel *Mytilus edulis:* no evidence for temperature compensation. Mar. Ecol. Prog. Ser. 305, 147–152. 10.3354/meps305147

[B32] Koester J. Dieringer N. Mandelbaum D. E. (1979). Cellular neuronal control of molluscan heart. Amer. Zool. 19, 103–116. 10.1093/icb/19.1.103

[B33] Kramer K. J. M. Foekema E. M. (2001). The ‘Musselmonitor®’ as biological early warning system - the first decade, in Biomonitors and Biomarkers as Indicators of Environmental Change 2 - A Handbook, eds Butterworth F. M. Gunatilaka A. Gonsebatt M. E. (Springer), 59–87. Available online at: http://www.springer.com/gp/book/9780306463877

[B34] Kubes P. Cain S. M. Chapler C. K. (1989). Neural regulation of canine skeletal muscle blood flow during hypoxic hypoxia. Am. J. Physiol. 257, H1581–H1586. 2589512 10.1152/ajpheart.1989.257.5.H1581

[B35] Liebeswar G. Goldman J. E. Koester J. Mayeri E. (1975). Neural control of circulation in Aplysia. III. Neurotransmitters. J. Neurophysiol. 38, 767–779. 1159464 10.1152/jn.1975.38.4.767

[B36] Liu C. C. Li L. Lam Y. W. Siu C. W. Cheng S. H. (2016). Improvement of surface ECG recording in adult zebrafish reveals that the value of this model exceeds our expectation. Sci. Rep. 6, 25073. 10.1038/srep25073 27125643 PMC4850402

[B37] Lowe G. A. (1974). Effect of temperature change on the heart rate of *Crassostrea gigas* and *Mya arenaria* (Bivalvia). Proc. Malac. Soc. Lond. 41, 29–36.

[B38] Mann R. (1979). Some biochemical and physiological aspects of growth and gametogenesis in *Crassostrea gigas* and *Ostrea edulis* grown at sustained elevated temperatures. J. Mar. Biol. Assoc. UK 59, 95–110. 10.1017/S0025315400046208

[B39] Mayeri E. Koester J. Kupfermann I. Liebeswar G. Kandel E. R. (1974). Neural control of circulation in Aplysia. I. Motoneurons. J. Neurophysiol. 37, 458–475. 4363777 10.1152/jn.1974.37.3.458

[B40] Mortola J. P. Marinescu D. C. Pierre A. Artman L. (2012). Metabolic and heart rate responses to hypoxia in early chicken embryos in the transition from diffusive to convective gas transport. Respir. Physiol. Neurobiol. 181, 109–117. 10.1016/j.resp.2012.02.002 22366866

[B41] Mortola J. P. Wills K. Trippenbach T. Al A. K. (2010). Interactive effects of temperature and hypoxia on heart rate and oxygen consumption of the 3-day old chicken embryo. Comp. Biochem. Physiol. A Mol. Integr. Physiol. 155, 301–308. 10.1016/j.cbpa.2009.11.003 19914389

[B42] Newell R. C. Branch G. M. (1980). The influence of temperature on the maintenance of metabolic energy balance in marine invertebrates. Adv. Mar. Biol. 17, 329–396. 10.1016/S0065-2881(08)60304-1

[B43] Peltier J.-C. A. (1834). Nouvelles experiences sur la caloricite des courants electriques. Ann. Chim. Phys. 56, 371–386.

[B44] Pickens P. E. (1965). Heart rate of mussels as a function of latitude, intertidal height, and acclimation temperature. Physiol. Zool. 38, 390–405. 10.1086/physzool.38.4.30152416

[B45] Polymeropoulos E. T. Plouffe D. Leblanc S. Currie S. Elliott N. G. Frappell P. B. (2014). Growth hormone transgenesis and polyploidy increase metabolic rate, alter the cardiorespiratory response and influence HSP expression to acute hypoxia in Atlantic salmon *(Salmo salar)* yolk-sac alevins. J. Exp. Biol. 217, 2268–2276. 10.1242/jeb.098913 24675560

[B46] Quayle D. B. (1969). Pacific Oyster Culture in British Columbia. Ottawa: Fisheries Research Board of Canada.

[B47] Rahbar S. Pan W. Jonz M. G. (2016). Purinergic and cholinergic drugs mediate hyperventilation in zebrafish: evidence from a novel chemical screen. PLoS ONE 11:e0154261. 10.1371/journal.pone.0154261 27100625 PMC4839714

[B48] Ritto P. A. Alvarado-Gil J. J. Contreras J. G. (2005). Scaling and wavelet-based analyses of the long-term heart rate variability of the Eastern Oyster. Physica A. 349, 291–301. 10.1016/j.physa.2004.10.020

[B49] Ritto P. A. Contreras J. G. Alvarado-Gil J. J. (2003). Monitoring of heartbeat by laser beam reflection. Meas. Sci. Technol. 14, 317–322. 10.1088/0957-0233/14/3/310

[B50] Ritto P. A. Vera D. Contreras J. G. Alvarado-Gil J. J. (2001). Study of the heartbeat of an invertebrate during long periods. American Institute of Physics. Med. Phys. Fifth Me. Symp. CP 593, 170–175. 10.1063/1.1420482

[B51] Ruckelshaus M. Doney S. C. Galindo H. M. Barry J. P. Chan F. Duffy J. E. (2013). Securing ocean benefits for society in the face of climate change. Mar. Policy 40, 154–159. 10.1016/j.marpol.2013.01.009

[B52] Sacha J. Barabach S. Statkiewicz-Barabach G. Sacha K. Muller A. Piskorski J.. (2013a). How to strengthen or weaken the HRV dependence on heart rate–description of the method and its perspectives. Int. J. Cardiol. 168, 1660–1663. 10.1016/j.ijcard.2013.03.038 23578892

[B53] Sacha J. Sobon J. Sacha K. Barabach S. (2013b). Heart rate impact on the reproducibility of heart rate variability analysis. Int. J. Cardiol. 168, 4257–4259. 10.1016/j.ijcard.2013.04.160 23680595

[B54] Sandercock G. R. Brodie D. A. (2006). The role of heart rate variability in prognosis for different modes of death in chronic heart failure. Pacing Clin. Electrophysiol. 29, 892–904. 10.1111/j.1540-8159.2006.00457.x 16923007

[B55] Skirtun M. Sahlqvist P. Vieira S. (2013). Australian fisheries statistics 2012. Canberra, November CC BY 3.0, Australian Bureau of Agricultural and Resource Economics and Sciences. Canberra: FRDC project 2010/208.

[B56] Tarkiainen T. H. Timonen K. L. Tiittanen P. Hartikainen J. E. Pekkanen J. Hoek G.. (2005). Stability over time of short-term heart rate variability. Clin. Auton. Res. 15, 394–399. 10.1007/s10286-005-0302-7 16362542

[B57] Tarvainen M. P. Niskanen J. P. Lipponen J. A. Ranta-Aho P. O. Karjalainen P. A. (2014). Kubios HRV–heart rate variability analysis software. Comput. Methods Programs Biomed. 113, 210–220. 10.1016/j.cmpb.2013.07.024 24054542

[B58] Theuerkauf S. Lipcius R. (2016). Quantitative validation of a habitat suitability index for oyster restoration. Front. Mar. Sci. 3:64 10.3389/fmars.2016.00064

[B59] The WorldFish Center (2011). Blue Frontiers: Managing the Environmental Costs of Aquaculture. Policy Brief No. 2011-24, 1-12 (Penang).

[B60] Thireau J. Zhang B. L. Poisson D. Babuty D. (2008). Heart rate variability in mice: a theoretical and practical guide. Exp. Physiol 93, 83–94. 10.1113/expphysiol.2007.040733 17911354

[B61] Trueman E. R. Blatchford J. G. Jones H. D. Lowe G. A. (1973). Recordings of the heart rate and activity of molluscs in their natural habitat. Malacologia 14, 377–383.

[B62] Trueman E. R. Lowe G. A. (1971). The effect of temperature and littoral exposure on the heart rate of a bivalve mollusc, *Isognomon alatus*, in tropical conditions. Comp. Biochem. Physiol. A 38, 555–564. 10.1016/0300-9629(71)90122-8

[B63] Tzeng Y. C. Larsen P. D. Galletly D. C. (2003). Cardioventilatory coupling in resting human subjects. Exp. Physiol. 88, 775–782. 10.1113/eph8802606 14603377

[B64] Veitch F. P. (1974). The preparation of “shell windows” in oysters. Mar. Chem. 2, 65–68. 10.1016/0304-4203(74)90007-3

[B65] Walne P. R. Spencer B. E. (1974). Experiments on the growth and food conversion efficiency of the spat of *Ostrea edulis* L. in a recirculatory system. J. Cons. Int. Explor. Mer. 35, 303–318. 10.1093/icesjms/35.3.303

[B66] Widdows J. (1973). Effect of temperature and food on the heart beat, ventilation rate and oxygen uptake of *Mytilus edulis*. Mar. Biol. 20, 269–276. 10.1007/BF00354270

